# Veno-Venous Surgical Bypass for Central Vein Occlusion in a Child: The First in the Literature

**DOI:** 10.1016/j.ejvsvf.2023.05.009

**Published:** 2023-05-19

**Authors:** Karthigesu Aimanan, Mang Ning Ong, Kean Leong Koay, Caroline Yin Eng Siew, Firdaus Hayati, Hafizan Tajri

**Affiliations:** a Department of Vascular Surgery, Hospital Serdang, Ministry of Health Malaysia, Kajang, Selangor, Malaysia; b Department of Paediatrics, Hospital Tuanku Ja'afar Seremban, Ministry of Health Malaysia, Seremban, Negeri Sembilan, Malaysia; c Department of Surgery, Faculty of Medicine and Health Sciences, Universiti Malaysia Sabah, Kota Kinabalu, Sabah, Malaysia

**Keywords:** Arteriovenous fistula, Veno-venous bypass, ESRF in Children, Central venous occlusion, Peritoneal dialysis

## Abstract

**Introduction:**

During the past two decades, the incidence of chronic kidney disease (CKD) in children worldwide has steadily increased and, even in children, native arteriovenous fistula (AVF) remains the access of choice. Nevertheless, maintaining a well functioning fistula is limited by central venous occlusion due to the widespread use of central venous access devices before AVF creation.

**Report:**

A 10 year old girl with end stage renal failure dialysing through a left brachiocephalic fistula presented with left upper limb and facial swelling. She had previously exhausted the option of ambulatory peritoneal dialysis for recurrent peritonitis. A central venogram showed occlusion at the left subclavian vein, which was not amenable for angioplasty through either an upper limb or femoral approach. Given the precious fistula with concomitant worsening venous hypertension, an ipsilateral axillary vein to external iliac vein bypass was performed. Subsequently, her venous hypertension was significantly resolved. This report is the first in English literature on this surgical bypass in a child with central venous occlusion.

**Discussion:**

Central venous stenosis or occlusion rates are rising due to extensive central venous catheter use in the paediatric population with end stage renal failure. In this report, an ipsilateral axillary vein to external iliac vein bypass was used successfully as a safe temporary option to maintain AVF. Ensuring a high flow fistula pre-operatively and continued antiplatelet post-operatively will allow longer patency of the graft.

## Highlights

• Central venous stenosis or occlusion is rising due to the widespread use of central venous catheters in the paediatric population with end stage renal failure.
• The central venous catheter is being used widely in children compared with arteriovenous fistula due to technical difficulty limited by small vessel diameter and low arterial flow rates.
• Successful use of an axillo-external iliac vein bypass as a safe option to maintain an arteriovenous fistula.

## Introduction

During the past two decades, the incidence of chronic kidney disease (CKD) in children has steadily increased and, even in children, native arteriovenous fistula (AVF) remains the access of choice.^1^^,^^2^ The rising rates of end stage renal failure (ESRF) among children illustrate the importance of knowledge about managing access related problems among clinicians. Early initiation of dialysis through a central venous catheter contributes to the risk of central venous occlusion in the future. Unlike in adults, maintaining a functioning AVF on the central venous occlusion side in children is a considerable challenge. This is the report of a 10 year old girl with ESRF secondary to steroid resistant nephrotic syndrome who presented with central vein stenosis; management plans are also discussed.

## Report

**Figure 1:**
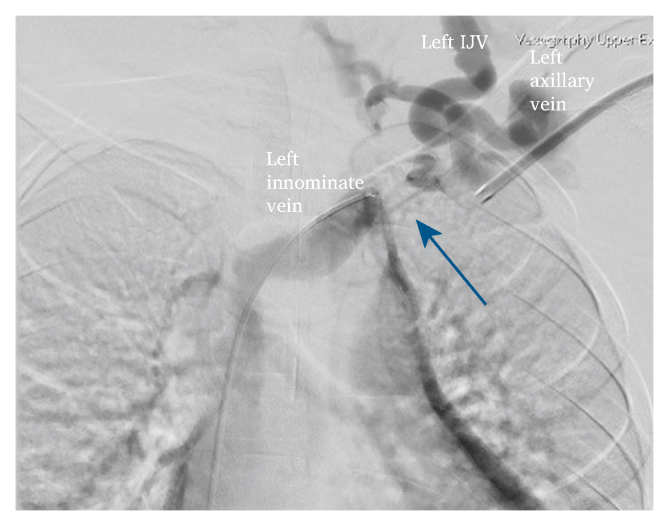
Left subclavian vein occlusion (blue arrow) demonstrated by venogram through the left arm and right femoral approach. IJV = internal jugular vein. (For interpretation of the references to color in this figure legend, the reader is referred to the Web version of this article).

**Figure 2:**
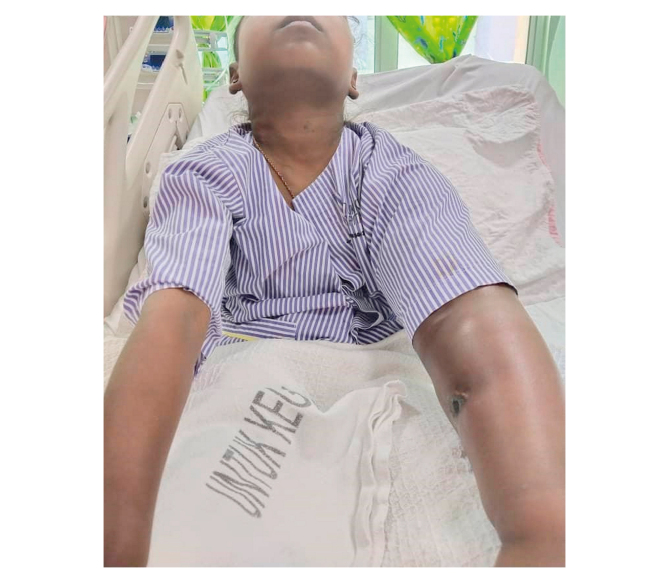
Ulcers on the left arm and forearm due to severe venous hypertension.

**Figure 3:**
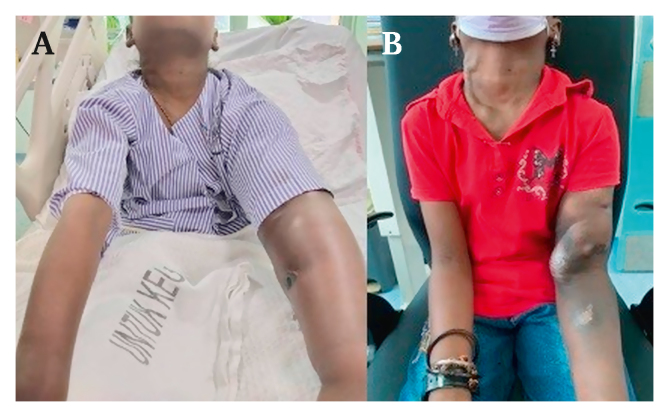
(A) Marked swelling of left upper limb prior to vein–vein bypass. (B) Significant reduction of left upper limb swelling a month after surgery.

A 10 year old girl with ESRF secondary to steroid resistant nephrotic syndrome presented to the vascular outpatient clinic with progressive left upper limb swelling. A left brachiocephalic fistula (BCF) had been created three years before and had functioned well to date. Her left upper limb swelling had started a year previously, and a conventional venogram confirmed left central vein stenosis (Fig. 1). An attempt at angioplasty failed due to occlusion at the left subclavian vein; then she was managed conservatively. Her venous hypertension became severe throughout the year, and she developed ulcers on the arm and forearm (Figs. 2 and 3A). Bilateral radial pulses were 2+. She was on continuous ambulatory peritoneal dialysis before creation of the AVF and had Tenckoff removal due to recurrent peritonitis. She also had bilateral internal jugular vein catheters, each lasting for two months, which were subsequently removed on the maturation of the AVF. At a review in the clinic, her BCF flow rate was 2.4 L/min. The patient and parents were then counselled regarding vein–vein bypass for treating venous hypertension secondary to central venous occlusion and maintaining her AVF.

An ipsilateral axillary vein to external iliac vein bypass using an 8 mm × 60 cm supported polytetrafluoroethylene graft (Atrium Advanta VS, Hudson, NY, USA) was performed. The left axillary vein was exposed via an infraclavicular incision and the left external iliac vein via an extraperitoneal approach. The graft was tunnelled inferior to the pectoralis minor at the proximal part into the axilla. Subsequently, it was tunnelled subcutaneously along the midaxillary line and lateral abdomen from the axillary vein to the ipsilateral external iliac vein. The proximal and distal anastomoses were done using an end (graft) to side (native vein) technique. The intra-operative course was uneventful. On the same day, the patient's left upper limb swelling improved immediately. Her dialysis was resumed through the left BCF the next day and she was discharged with a single antiplatelet. In the clinic, her BCF flow rate was 1.8 L/min, the graft flow rate was 1.2 mL/min, and the upper limb swelling had markedly improved (Fig. 3B). The patient's parents were advised to be highly vigilant regarding pressure changes during dialysis and to return early if a rise in venous pressure was noticed. Twelve months later, her bypass graft remains patent.

### Discussion

Rising ESRF numbers among children highlight the importance of knowledge among clinicians about the existing armamentarium on dialysis access in children. In children requiring renal replacement therapy, renal transplantation is the preferred choice worldwide due to the superior quality of life it offers. However, if pre-emptive transplantation is unavailable, peritoneal dialysis or haemodialysis are potential alternatives. Haemodialysis through an AVF has more advantages than a central venous catheter (CVC) in terms of its greater longevity and lower rate of infection. Despite this, the central venous catheter is being used more widely in children than AVF due to the technical difficulties when limited by small vessel diameter and low arterial flow rates.

The complications of a CVC include complete occlusion or stenosis of the central veins. CVC dependence of more than six months is a significant risk factor for developing central vein stenosis or occlusion.^3^ Veins included in a central venous occlusion are the intrathoracic part of the internal jugular vein, the subclavian veins, the brachiocephalic veins, and the superior vena cava. Pathophysiology is explained by repeated trauma related to catheter movement against the vein wall and inflammatory changes secondary to a foreign body reaction. The management of central venous stenosis ranges from clinical observation to radiological intervention or surgery. Asymptomatic stenosis generally requires no intervention. Symptomatic patients present with unilateral upper limb swelling and dilated chest veins due to high venous pressure and blood flow from the functioning fistula. In severe cases, patients present with signs of venous hypertension, such as ulcers and gangrene of the digits.

Repeated central venoplasty is the intervention of choice in symptomatic cases to relieve obstructive symptoms and also ensure the continued patency of the fistula. A surgical procedure is the next step in patients for whom the endovascular approach has failed. Unfortunately, there are very few publications on managing central venous stenosis in the paediatric population. de Buys Roessingh et al. reported a successful recanalisation of superior vena cava (SVC) stenosis via an endovascular femoral approach in two patients under one year of age.^4^ Rinat et al. reported the successful management of three of four patients with central venous stenosis.^5^ A total of two patients had the AVF ligated, and the central venous stenosis symptoms slowly subsided. In a patient with SVC occlusion, an SVC vein to the right atrium bypass was created. This surgery has higher morbidity than an axillary vein to external iliac vein bypass, as was performed on this patient, due to the avoidance of sternotomy.^5^ Another option is to perform a first rib resection (open or video assisted or robot assisted) followed by venous angioplasty.^6^ It can provide an excellent intra-operative visualisation and produce a shorter hospital stay as the main advantages of a minimally invasive approach.

Almost all the evidence of surgical vein–vein bypasses is from adult case series and reports. The available options for a surgical vein–vein bypass include outflow to an ipsilateral unobstructed external or internal jugular vein; contralateral subclavian or internal jugular vein; lower extremity venous outflow, such as the femoral or popliteal vein; and intrathoracic or intra-abdominal vena cava or right atrium. Dammers et al. have reported one year primary patency of 75% among the 10 adult patients with surgical reconstruction for central venous occlusion.^7^ However, the limitations of the surgical bypass – such as general anaesthesia related risks, graft infection, and tunnelling related haematoma – mean this option carries high initial risks.^7^

A common concern when using any synthetic graft in the paediatric population is the inability of the graft to elongate with the growth spurts of the patient. Growth retardation among children with end stage renal failure is well known; thus, it was decided to proceed with the surgical bypass using a graft. Alternative options, such as a great saphenous vein substituting synthetic graft for a vein–vein bypass, were unacceptable due to the smaller calibre of the conduit, which entails a high probability of thrombosis. However, graft extension bypass is a potential future option if a problem rises due to the child's growth.

Maintaining the bypass graft's patency is important while awaiting a definitive treatment such as a renal transplant. Saravanan et al. have reported a few factors to ensure a successful outcome of a vein to vein bypass graft, such as a high pre-operative fistula flow rate (>1500 mL/min), long term antiplatelets, and ensuring a graft flow rate of more than 1000 mL/min during follow up in order to maintain a well functioning graft.^8^ However, there needs to be more information in other similar series regarding the benefit of antiplatelet therapy and duration.

In conclusion, central venous stenosis or occlusion rates are rising due to extensive CVC use in the paediatric population with end stage renal failure. This report shows successful use of axillary vein to external iliac vein bypass as a safe option to maintain an AVF in central vein occlusion. Ensuring a high flow fistula pre-operatively and continued antiplatelets post-operatively will ensure the longer patency of the graft.

## Conflicts of interest

None.

## Funding

None.
